# Predicting mortality in adult patients with sepsis in the emergency department by using combinations of biomarkers and clinical scoring systems: a systematic review

**DOI:** 10.1186/s12873-021-00461-z

**Published:** 2021-06-13

**Authors:** Kirby Tong-Minh, Iris Welten, Henrik Endeman, Tjebbe Hagenaars, Christian Ramakers, Diederik Gommers, Eric van Gorp, Yuri van der Does

**Affiliations:** 1 Department of Emergency Medicine, Erasmus University Medical Center, Rotterdam, Postbus 2040, 3000 CA Rotterdam, The Netherlands; 2 Department of Intensive Care, Erasmus University Medical Center, Rotterdam, the Netherlands; 3 Department of Clinical Chemistry, Erasmus University Medical Center, Rotterdam, the Netherlands; 4 Department of Internal Medicine, Erasmus University Medical Center, Rotterdam, the Netherlands; 5 Department of Viroscience, Erasmus University Medical Center, Rotterdam, the Netherlands

**Keywords:** Sepsis, Biomarkers, Emergency department, Prediction model

## Abstract

**Background:**

Sepsis can be detected in an early stage in the emergency department (ED) by biomarkers and clinical scoring systems. A combination of multiple biomarkers or biomarker with clinical scoring system might result in a higher predictive value on mortality. The goal of this systematic review is to evaluate the available literature on combinations of biomarkers and clinical scoring systems on 1-month mortality in patients with sepsis in the ED.

**Methods:**

We performed a systematic search using MEDLINE, EMBASE and Google Scholar. Articles were included if they evaluated at least one biomarker combined with another biomarker or clinical scoring system and reported the prognostic accuracy on 28 or 30 day mortality by area under the curve (AUC) in patients with sepsis. We did not define biomarker cut-off values in advance.

**Results:**

We included 18 articles in which a total of 35 combinations of biomarkers and clinical scoring systems were studied, of which 33 unique combinations. In total, seven different clinical scoring systems and 21 different biomarkers were investigated. The combination of procalcitonin (PCT), lactate, interleukin-6 (IL-6) and Simplified Acute Physiology Score-2 (SAPS-2) resulted in the highest AUC on 1-month mortality.

**Conclusion:**

The studies we found in this systematic review were too heterogeneous to conclude that a certain combination it should be used in the ED to predict 1-month mortality in patients with sepsis. Future studies should focus on clinical scoring systems which require a limited amount of clinical parameters, such as the qSOFA score in combination with a biomarker that is already routinely available in the ED.

**Supplementary Information:**

The online version contains supplementary material available at 10.1186/s12873-021-00461-z.

## Background

Sepsis is a life threatening condition, and is the leading cause of in-hospital mortality in Europe [1]. Early detection of sepsis is essential to timely start appropriate treatment [2, 3]. Early stage sepsis, in patients with a suspected infection, is often undiagnosed, causing a delay in treatment and increased mortality [4, 5]. The emergency department (ED) is often the first setting during hospital stay where patients with a suspected infection are systematically evaluated, where early stages of sepsis can be detected. However, there is a limited timeframe in the ED in which decisions about treatment and patient disposition must be made. Identifying patients in the ED with a high risk of mortality is important, not only to start antibiotic treatment early, but also to decide if patients require admission, high level care and monitoring.

Multiple organ systems and pathways are involved in the pathophysiology of sepsis [6]. After a microorganism infects the body, multiple immune responses are activated [7]. Different immune cells are activated, which express a series of membrane receptors, endothelial and tissue factors are released, and the complement system is activated [8]. In sepsis, this immune response is dysregulated and excessive, ultimately resulting in multi-organ failure [9]. This response involves dysregulation by both hyperinflammation and immune suppression [10, 11]. In these different stages of sepsis, different cytokines, peptides and other signaling molecules are elevated and can be detected in the bloodstream as biomarkers.

Clinical scoring systems, which are often used to detect sepsis, rely on vital parameters. Clinical scoring systems should require only a limited amount of clinical parameters to be useful in the ED to rapidly assess the severity of disease. Different clinical scoring systems have been validated for use in the ED, including the Quick Sequential Organ Failure Assessment (qSOFA) [9], the Mortality in Emergency Department Sepsis (MEDS) [12] score and National Early Warning Score (NEWS) [13]. (Supplemental Table 1) These clinical scoring systems mostly rely on the use of abnormal vital parameters. However, when vital parameters are abnormal, the patient might already be in an advanced stage of sepsis. Using biomarkers to detect sepsis, early stages of sepsis could be detected before vital signs turn abnormal. Adding biomarkers to clinical scoring systems might therefore improve these clinical scoring systems. A large variety of biomarkers in patients with sepsis have been studied. Pierrakos et al. reviewed the literature on biomarkers in sepsis in 2010 and in 2020 and found that there are over 100 different biomarkers studied, of which none have made it to clinical practice except C-reactive protein (CRP) and procalcitonin (PCT) [14, 15]. Even 10 years after their initial study, no specific other biomarker was identified as “most promising” biomarker [15]. The authors concluded that a combination of several biomarkers may be more effective. Several studies support this claim and show that combining biomarkers with clinical scoring systems or combining multiple biomarkers result in a more accurate prediction of mortality in patients with infectious diseases in the ED. [16, 17]

With the large number of biomarkers already studied, the potential number of combinations of biomarkers and clinical scoring systems is even greater.

The goal of this review is to systematically assess the available literature on combinations of biomarkers and clinical scorings systems in patients with sepsis in the ED to predict 1-month mortality.

## Methods

We conducted this systematic review and reported this following the Preferred Reporting Items for Systematic Reviews and Meta-analyses (PRISMA) guidelines [18]. The study was registered on PROSPERO, the register for systematic reviews under reference number 165580.

### Literature search

We performed a systematic search of literature by an information specialist using MEDLINE, EMBASE and Google Scholar. The search included articles in English published up to June 2020. We started with a broad search using both infectious diseases and sepsis in the initial search in combination with biomarkers and ED. The full search strategy can be found in the Appendix.

### Outcome definitions

Biomarkers were considered as any laboratory blood test performed in the ED. A clinical scoring system was defined as any scoring system using a combination of patient characteristic with or without laboratory testing used for prognostic purpose. We did not use a single definition of sepsis in this review. Sepsis criteria changed multiple times in the previous decades and we did not want to miss any potential useful article in our initial search strategy We used 1-month mortality as outcome, which was defined as either 28 or 30-day mortality.

### Study selection

**Fig. 1:**
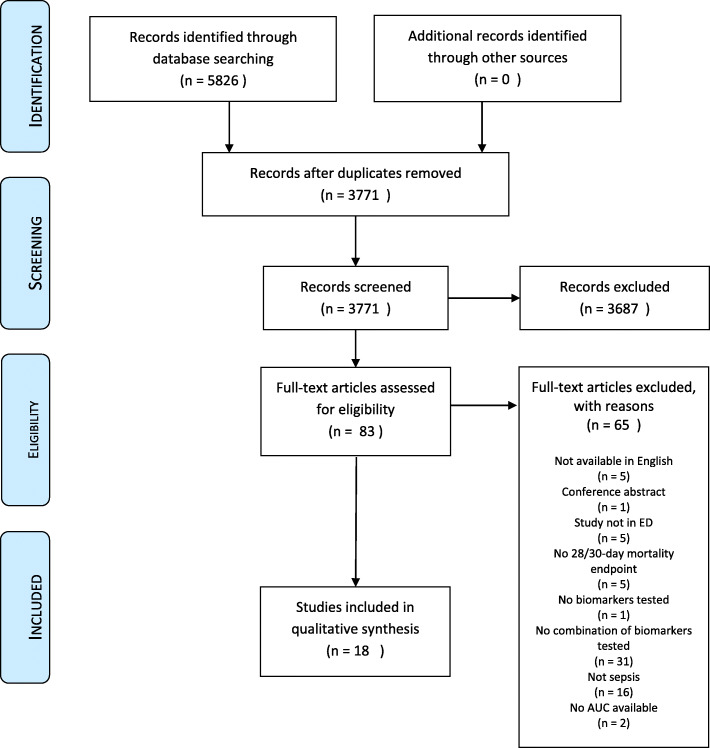
Flow chart of included articles

After the initial search, two reviewers independently screened the studies by title and abstract from each other. The results were compared and discrepancies were resolved by discussion. If no consensus was achieved, a third reviewer acted as referee. The remaining studies were screened on inclusion and exclusion criteria using the full text (Fig. 1).

### Selection criteria

During title and abstract screening, articles were included if they evaluated at least one biomarker or clinical scoring system in any infectious disease. During full text screening, articles were included if they evaluated at least one biomarker combined with another biomarker or clinical scoring system and reported the prognostic accuracy on 28 or 30 day mortality by AUC in patients with sepsis. Other measurements of the studied prediction models were recorded, such as sensitivity, specificity and Hosmer-Lemeshow test statistic, if they were reported by the authors. However, articles were not excluded if these values were missing.

Studies in children and studies of which the full text was not available in English were excluded.

### Data collection and quality assessment

Data was extracted in a predefined spreadsheet, which included the biomarkers and clinical scoring system used, AUC of the combination of biomarkers and clinical scoring system, age, inclusion and exclusion criteria and moment of blood collection. The quality of each study was assessed using Prediction model Risk Of Bias ASsessment Tool (PROBAST) [19]. This risk of bias was assessed and reported during the PROBAST assessment.

## Results

We found 5826 articles after conducting our search in PubMed, EMBASE, Medline Ovid, Web of Science, Cochrane Central and Google Scholar. After removal of duplicates, 3771 articles remained. These articles were screened on title and abstract, after which 83 articles were included for full text screening. After full text screening, 65 articles were excluded. This resulted in 18 articles included for final data synthesis. (Fig. 1).

In the 18 articles that were included in this systematic review, a total of 35 combinations of biomarkers and clinical scoring systems were studied of which 33 unique combinations. In total, seven different clinical scoring systems and 21 different biomarkers were investigated. (Table 1).

**Table 1:** Overview of included studies

Author, year	Sepsis criterion	Number of patients	Number of deaths	Biomarker 1	Biomarker 2	Biomarker 3	Clinical score 1	Clinical score 2	AUC	Hosmer-Lemeshow	Sensitivity	Specificity	PPV	NPV
Yu, 2019 [20]	Signs of systemic infection	1318	178	PCT			qSOFA		0.73					
				CRP			qSOFA		0.69					
Yamamoto, 2015 [21]	Blood cultures drawn as surrogate marker of suspected sepsis	1262	106	CRP			CURB65		0.77	0.607				
Liu, 2013 [22]	Sepsis-2	859	Unknown	Presepsin			MEDS		0.731					
				Presepsin			APACHE-2		0.734					
Zhang, 2014 [23]	Sepsis-2	680	137	Copeptin			MEDS		0.851					
				Cortisol			MEDS		0.833					
				PCT	Cortisol	Copeptin	MEDS		0.891					
Chen, 2014 [24]	Sepsis-2	680	178	Lactate			MEDS		0.81					
				Lactate			APACHE-2		0.81					
				Lactate			SOFA		0.82					
Yin, 2013 [25]	Sepsis-2	680	225	sTM			MEDS		0.805		74.2	71.9	56.6	84.9
Zhao, 2018 [26]	Sepsis-2	655	126	PCT	sPD-1		MEDS		0.843	0.824	81.6	83.4	71.7	89.8
				PCT			MEDS		0.792	0.631				
				sPD-1			MEDS		0.829	0.892				
Niño, 2017 [27]	Sepsis-2	563	68	TIMP1	MMP9		CHARLSON	SOFA	0.838	0.2449				
Zhao, 2013 [28]	Sepsis-2	501	134	PCT			MEDS		0.813		67.2	81.2	56.6	87.1
Zhang, 2016 [29]	Sepsis-2	480	183	IgE			APACHE-2		0.8					
				IgE			SOFA		0.781					
				IgE			MEDS		0.89					
Wang, 2014 [30]	Sepsis-2	480	137	NGAL			MEDS		0.858					
				TIMP-1			MEDS		0.882					
				PCT			MEDS		0.782					
Henning, 2019 [31]	Sepsis-2	314	31	Angiopoientin-2	IL-6				0.72					
Chen, 2014 [32]	Sepsis-2	295	78	H-FABP			MEDS		0.853					
				H-FABP			APACHE-2		0.826					
				Troponine-I			APACHE-2		0.811					
				Troponine-I			MEDS		0.825					
Duplessis, 2018 [33]	Sepsis-2	203	13	Nucleosomes			APACHE-2		0.84					
				Cell free DNA			APACHE-2		0.81					
Kofoed, 2008 [34]	Sepsis-2	161	9	suPAR	sTREM-1		SAPS-2		0.89					
				suPAR			SAPS-2		0.93					
Viallon, 2008 [35]	Sepsis-2	131	23	PCT	IL-6	Lactate	SAPS-2		0.939					
Carpio, 2015 [36]	Sepsis-2	114	48	Presepsin			MEDS		0.878					
Song, 2019 [37]	Sepsis-3	113	13	IL-6	PTX3				0.637		82.7	71.1		

Scoring systems. *qSOFA* quick Sequential Organ Failure Assessment, *MEDS* Mortality in Emergency Department Sepsis, *APACHE-2* Acute Physiologic Assessment and Chronic Health Evaluation II, *SOFA* Sequential Organ Failure Assessment, *SAPS-2* Simplified Acute Physiology Score 2

*PCT* Procalcitonin, *CRP* C-reactive protein, *sTM* soluble thrombomodulin, *sPD-1* soluble programmed death 1, *TIMP-1* Tissue inhibitor of metalloproteinase-1, *MMP9* Matrix metallopeptidase 9, *NGAL* Neutrophil gelatinase-associated lipocalin, *IL-6* interleukin-6, *H-FABT* heart fatty acid binding protein, *suPAR* soluble urokinase-type plasminogen activator receptor, *PTX3* Pentraxin 3

The Mortality in Emergency Department Sepsis (MEDS) score was the most used clinical scoring system, which was studied in 9 articles. The second most commonly used scoring system was the Acute Physiology and Chronic Health Evaluation II (APACHE II) score, which was studied in 6 articles. The most commonly studied biomarker was PCT, which was studied in 7 articles. The combination of PCT with the MEDS score was the most studied combination of biomarker and clinical score and was studied in 3 different articles. There were no other combinations of biomarkers that were used by more than a single article.

The number of patients included in the studies ranged from 114 to 1318. The AUC of the combinations of biomarkers and clinical scoring systems ranged from 0.637 to 0.939. The highest AUC was achieved by the combination of Simplified Acute Physiology Score (SAPS II), PCT, lactate and interleukine-6 (IL-6), which yielded an AUC of 0.939 by Viallon et al. [35], followed by the combination of SAPS-2 and soluble urokinase-type plasminogen activator receptor (suPAR) with an AUC 0.930 by Kofoed et al. [34]

Different inclusion criteria were used to classify patients as having sepsis. The most common criteria used were two SIRS criteria in combination with an infection, used by eight articles [25, 28, 31–36]. The second most used inclusion criterion was the 2001 International Sepsis Definitions [38], which was used by seven articles [22–24, 27, 29, 30, 32]. One article used the sampling of blood cultures as inclusion criterion [21]. One article included patients with symptoms of systemic infection in which PCT or blood cultures were taken within 24 h of admission [20]. One article used the Sepsis-3 definition [9] as inclusion criterion [37].

Eight studies reported other characteristics of the studied prediction models besides the AUC. The Hosmer-Lemeshow statistic was reported in five studies and ranged from 0.245 to 0.892. Four studies reported sensitivity, specificity, negative predictive value or positive predictive value and preselected cut-off values.

### PROBAST quality assessment

The quality assessment using the PROBAST criteria can be found in Table 2 and the extended checklist with the signaling questions used in Supplemental Table 2 and 3. The majority of the studies were at risk of bias: 12 out of 18 studies [22, 23, 25–28, 30, 31, 33–37] scored high at any of the items of the PROBAST checklist.

**Table 2:** PROBAST assessment

Author, year	Domain 1: participants	DOMAIN 2: Predictors	DOMAIN 3: Outcome	DOMAIN 4: Analysis	Overal risk of bias
Yu, 2019	Low	Low	Low	Low	Low
Yamamoto, 2015	Low	Low	Low	Low	Low
Liu, 2013	Low	Low	Low	High	High
Zhang, 2014	Low	Low	Low	High	High
Chen, 2014	Low	Low	Low	Low	Low
Yin, 2013	Low	Low	Low	High	High
Zhao, 2018	High	Low	Low	Low	High
Niño, 2017	Low	Low	Low	High	High
Zhao, 2013	High	Low	Low	Low	High
Zhang, 2016	Low	Low	Low	Low	Low
Wang, 2014	Low	Low	Low	High	High
Henning, 2019	Low	Low	Low	High	High
Chen, 2019	Low	Low	Low	Low	Low
Duplessis, 2018	Low	Low	Low	High	High
Kofoed, 2008	Low	Low	Low	High	High
Viallon, 2008	Low	Low	Low	High	High
Carpio, 2015	Low	Low	Low	High	High
Song, 2019	Low	Low	Low	High	High

One study [26] was at risk of bias in the participant domain due to multiple exclusion criteria, leading to only a selected group of patients with sepsis enrolled in the study. There was no risk of bias in any of the studies in the domains predictors and outcome. In the domain analysis, 11 studies were considered at high risk of bias [22, 23, 25, 27, 28, 30, 31, 33–37]. The number of fatal cases was low in seven studies, leading to risk of overfitting of the studied prediction model [30, 31, 33–37]. Eleven studies did not report any missing data or did not report how missing data was handled [22, 23, 25, 27, 29, 30, 32, 34–37]. These items were not scored as high risk of bias, because all enrolled patients were included in the final analysis.

## Discussion

Biomarkers and clinical scoring systems help physicians to detect sepsis in an early stage in the ED. In this systematic review we investigated the combinations of both biomarkers and clinical scoring systems with biomarkers to predict 1-month mortality in patients with sepsis. We found 18 different studies in which 33 combinations of biomarkers and clinical scoring system were investigated. The combination of PCT, lactate, IL-6 and SAPS-2 resulted in the highest AUC on 1-month mortality [35]. Despite the high AUC found in this study, this specific combination has not been adopted in the latest guidelines for surviving sepsis [2]. The SAPS-2 score is a clinical scoring system, using four vital parameters, seven laboratory tests and four other patient characteristics and was originally developed for patients in the ICU or general wards to predict in-hospital mortality. Combining this clinical scoring system with another three biomarkers results in a total of 18 variables used to predict 30-day mortality in this study. This large amount of data needed for this combination results in only limited usefulness for clinical practice. Furthermore, this study enrolled 131 subjects, of which 23 died. Therefore, the high AUC found in this study may possibly be due to overfitting by using too many predictors in the multivariate logistic regression analysis [39, 40].

To introduce a new combination of biomarkers and clinical scoring system to clinical practice, the biomarker should be available as routine test in the clinical laboratory and available together with the standard laboratory tests. Furthermore, the clinical scoring system should require only a limited amount of variables, because of the limited timeframe in which patients stay in the ED. In our study, only a limited amount of articles met those criteria. Many articles investigated experimental biomarkers such as NGAL, cell free DNA or sPD-1, making these combinations less feasible for clinical practice. Following these criteria, only a few articles we found would qualify for potential use in clinical practice. Yamamoto et al. combined the CURB-65 score with CRP. CURB-65 was originally developed for disposition decisions in patients with pneumonia, but was in this study used in suspected sepsis patients. This resulted in an AUC of 0.77 on 1-month mortality. However, the authors concluded that adding CRP to the CURB-65 only had limited clinical value, because the CURB-65 score alone showed a similar predictive value. Yu et al. investigated the combination of PCT and the qSOFA and showed that adding PCT improved the performance of the qSOFA score. This was the only study using the most recent Sepsis-3 criteria and incorporating the qSOFA score. The qSOFA score is validated for early detection of adverse outcomes in patients with infectious diseases and requires only 3 vital parameters. Combining it with PCT, a biomarker that is already available as routine measurement in many ED, makes this combination practical to use in the ED.

We found four studies using PCT combined with another biomarker or clinical scoring system. PCT has been studied as biomarker for bacterial infections and disease severity in infectious diseases. PCT is the precursor of calcitonin and physiologically produced by thyroid cells. In bacterial infections it is also synthesized outside of the thyroid, and rises rapidly in systemic infections. It is often referred to as the biomarker with most potential of replacing or substituting CRP [41]. However, PCT has yet to establish a role in routine care in the ED. [42] Combining PCT with other biomarkers or clinical scoring systems, we found an increase in predictive value on 30-day mortality. From all available sepsis biomarkers, PCT is probably the most well-known among physicians in the ED. Combining PCT with clinical scoring systems, as done in many studies, might therefore be the key in being adopted as part of regular care.

Lactate is a product of anaerobic glycolysis and is often elevated in patients with sepsis. It has been adopted as criterion for septic shock in the Sepsis-3 definitions [9]. We found two studies using lactate in combination with other biomarkers or clinical scoring system, both with a high predictive value on 1-month mortality [32, 35]. Unlike many novel biomarkers, lactate is widely available as a standard measurement during the workup in the ED. Therefore, lactate is an important biomarker in assessing the severity of sepsis in the ED. IL-6 is an inflammatory cytokine and plays an important role in the early phase of sepsis [43]. However, the prognostic values of IL-6 are controversial due to the short window in which IL-6 rises and falls during inflammation and infection [44].

The SAPS-2 score was also used in combination with suPAR by Kofoed et al. [34], resulting in an AUC of 0.930, which was the second highest AUC we found in our study. These findings suggest that the SAPS-2 is a clinical scoring system with a high prognostic accuracy on 30-day mortality, although it has not been validated for assessing severity of disease in the ED. However, the limitation of an overfitted prediction model in a relatively small cohort is also present in the study of Kofoed et al., with only 161 patients enrolled of which 9 patients died. suPAR is a biomarker which has been investigated as general disease severity biomarker, mostly in the ED. A large study showed that suPAR is an accurate predictor of mortality, but does not influence disposition or clinical outcome when it was used in the ED. [45] In a meta-analysis, suPAR showed similar results as PCT in diagnosing sepsis [46].

The MEDS score was the most used clinical scoring system used in combination with biomarkers. The MEDS score is a risk prediction score specifically for patients with suspected sepsis in the ED. [12] It consists of nine items which can easily be scored in the ED setting and results in a total score, categorized in 5 groups, which corresponds to a certain mortality risk. The AUC of the MEDS score combined with different biomarkers ranged from 0.731 to 0.891, indicating a moderate to good predictive value on 30-day mortality. Other clinical scoring systems we found in combination with biomarkers were the APACHE-2 and SAPS-2 score. These clinical scoring systems are mainly developed for use in the ICU and general wards. Despite being accurate predictors of disease severity, these clinical scoring systems may be less feasible for use in the ED, due to their complexity and large number of clinical parameters needed. In a prospective study comparing different clinical scoring systems individually in the ED, the MEDS score resulted in an AUC of 0.94 on 30-day mortality, which was higher than the SOFA or PIRO score [47]. However, another study which also compares different clinical scoring systems concluded that the APACHE-2 score is superior to the MEDS and SOFA score [48].

Three studies investigated biomarkers which are otherwise known as hormones and other functional circulating peptides, including IgE, cortisol, cell free DNA and nucleosomes [23, 29, 33]. Zhang et al. [29] studied IgE in combination with the MEDS score and found that adding IgE to the MEDS score resulted in a higher AUC than the MEDS score alone. This study emphasizes the multifactorial entity of sepsis, hypothesizing that IgE either plays a role in general immune activation during sepsis or is a marker of cytokine regulation/dysregulation. Another study by Zhang et al. [23] investigated hormones and biomarkers from the hypothalamic–pituitary–adrenal axis and showed that cortisol and copeptin are associated with 30-day mortality and that combining these biomarkers with the MEDS score resulted in added value over using each biomarker individually. Cortisol has been identified as essential hormone in the immune response in sepsis and elevated levels of cortisol are associated with severity of sepsis [49]. Extracellular cell free DNA and nucleosomes, basic units of DNA packaging, reflect cellular apoptosis and are therefore tested as predictors of severity of sepsis in the study of Duplessis et al. [33] In this study the authors show that adding nucleosomes to the APACHE-2 score improved the AUC on predicting mortality. Adding cell free DNA to the APACHE-2 score did not result in a better predictive value. These studies emphasize that biomarkers originating from different pathways in sepsis can be used as predictor of disease severity.

### Limitations

Our study has several limitations. We acknowledge that there are many studies available in a similar setting and research field, which were not included in this review because these articles did not exactly match our inclusion criteria. We included articles that investigated the predictive value of biomarkers combined with clinical scoring systems on disease severity in sepsis. We used 1-month mortality as endpoint for severity of sepsis as this is the most commonly used endpoint in these kind of studies. However, there are many more biomarkers that have been investigated using other endpoints as marker of disease severity of sepsis. These endpoints to define severity of sepsis range from ICU admission to long term mortality. Despite the fact that these endpoints also are a surrogate marker of disease severity, these articles were not included because a comparison of these endpoints would not be possible.

The definition of sepsis has changed over time, which is also reflected by the different inclusion criteria used by the studies we found. Most studies used the sepsis criteria as defined in 2001 [38], and only a few studies used the latest Sepsis-3 criteria [9]. The majority of the studies we analyzed in this systematic review were published before 2016 and do not use the latest sepsis criteria. However, even after 2016, multiple studies still used the old sepsis criteria. These studies may have been designed and conducted before 2016, but this also emphasizes that it takes much time before new criteria are adopted in clinical practice.

All but three studies did not use predefined cut-off values for the biomarkers. This makes translating these results to clinical practice challenging. Using a predefined cutoff, categorizing the biomarker or clinical scoring system in a high or low risk category, is preferred when using such a system in practice.

In the PROBAST quality assessment, we considered the majority of included studies of high risk of bias. This was mainly due to the high risk of overfitting. The study population size of the included studies ranged from 114 to 1318. When there are less than 10 fatal cases per predictor, the risk of overfitting of prediction models is high, resulting in an unrealistically high AUC [40]. When also including clinical scoring systems, this problem is even bigger, since the clinical scoring system already consists of multiple predictors. The findings of our study can therefore not directly be translated into clinical practice and need to be validated in larger and external cohorts.

Conducting a meta-analysis to compare the outcomes of different studies would be preferred, but was not feasible. The variety in biomarkers and clinical scoring systems used was too large to compare one to another. We selected the use of an AUC as indicator of performance of the prediction models as inclusion criterion. Only a limited number of studies reported other qualities such as Hosmer-Lemshow statistic. Furthermore, the disease severity may have differed between the different studies at time of presentation at the ED. We did not systematically collect data on triage scores nor exact time or moment of biomarker measurement in the ED, because these details are often not reported. This would further limit comparison between different prediction models.

## Conclusion

The studies we found in this systematic review were too heterogeneous to conclude that a certain combination it should be used in the ED to predict 1-month mortality in patients with sepsis. The combination of PCT, IL-6, lactate and the SAPS-2 score had the highest AUC on 1-month mortality in patients with sepsis in the ED, but this finding may be overfitted and requires external validation. Future studies should focus on clinical scoring systems which require a limited amount of clinical parameters, such as the qSOFA score in combination with a biomarker that is already routinely available in the ED.

### Supplementary Information

**Additional file 1: Supplemental Table 1**: overview of commonly used clinical scoring systems for mortality prediction. **Supplemental table 2**: Extended PROBAST checklist. **Supplemental table 3**: Signaling questions used in PROBAST checklist.

## Data Availability

The datasets used and/or analysed during the current study are available from the corresponding author on reasonable request.

## Appendix A

**Complete search strategy.**

**embase.com**

(‘sepsis’/exp. OR ‘infection’/exp. OR pneumonia/exp. OR (sepsis OR septic* OR Bacteremia OR Bacteraemia OR fungemia OR fungaemia OR urosepsis OR infection* OR pneumonia* OR meningitis* OR meningoencephalitis*):ab,ti) AND (‘emergency ward’/exp. OR ‘emergency patient’/de OR ‘emergency health service’/de OR ‘hospital emergency service’/de OR ‘emergency care’/de OR ‘emergency physician’/de OR ‘emergency medicine’/de OR (((emergency) NEAR/6 (ward* OR department* OR room OR unit OR cent* OR patient* OR care OR healthcare OR physician* OR medicine)) OR ‘acute care’ OR ed):ab,ti) AND (‘biological marker’/exp. OR ‘marker’/de OR ‘molecular marker’/de OR ‘disease marker’/de OR ‘blood level’/de OR ‘lactate blood level’/de OR ‘protein blood level’/de OR (((biological* OR bio* OR inflammat* OR diagnos* OR molecul* OR disease) NEAR/3 marker*) OR biomarker* OR ((serum OR blood) NEAR/6 (concentrate* OR level* OR marker* OR lactate OR protein* OR elevat*))):ab,ti) NOT (‘case report’/de OR (case-report*):ti) NOT ([Conference Abstract]/lim) AND [English]/lim NOT ((juvenile/exp. OR pediatrics/exp. OR ‘newborn sepsis’/de OR ‘pediatric emergency medicine’/de OR (child* OR adolescen* OR infan* OR newborn* OR neonat*):ti) NOT (adult/exp. OR (adult*):ti)) NOT ([animals]/lim NOT [humans]/lim)

**Medline Ovid.**

(exp Sepsis/ OR exp. Infection/ OR exp. Pneumonia/ OR (sepsis OR septic* OR Bacteremia OR Bacteraemia OR fungemia OR fungaemia OR urosepsis OR infection* OR pneumonia* OR meningitis* OR meningoencephalitis*).ab,ti.) AND (Emergency Medical Services/ OR Emergency Service, Hospital/ OR Emergency Medicine/ OR (((emergency) ADJ6 (ward* OR department* OR room OR unit OR cent* OR patient* OR care OR healthcare OR physician* OR medicine)) OR acute care OR ed).ab,ti.) AND (exp Biomarkers/ OR blood.fs. OR (((biological* OR bio* OR inflammat* OR diagnos* OR molecul* OR disease) ADJ3 marker*) OR biomarker* OR ((serum OR blood) ADJ6 (concentrate* OR level* OR marker* OR lactate OR protein* OR elevat*))).ab,ti.) NOT (case reports/ OR (case-report*).ti.) AND english.la. NOT ((exp child/ OR exp. infant/ OR Pediatrics/ OR Neonatal Sepsis/ OR Pediatric Emergency Medicine/ OR (child* OR adolescen* OR infan* OR newborn* OR neonat*).ti.) NOT (exp adult/ OR (adult*).ti.)) NOT (exp animals/ NOT humans/)

**Web of science.**

TS = (((sepsis OR septic* OR Bacteremia OR Bacteraemia OR fungemia OR fungaemia OR urosepsis OR infection* OR pneumonia* OR meningitis* OR meningoencephalitis*)) AND ((((emergency) NEAR/5 (ward* OR department* OR room OR unit OR cent* OR patient* OR care OR healthcare OR physician* OR medicine)) OR “acute care” OR ed)) AND ((((biological* OR bio* OR inflammat* OR diagnos* OR molecul* OR disease) NEAR/2 marker*) OR biomarker* OR ((serum OR blood) NEAR/5 (concentrate* OR level* OR marker* OR lactate OR protein* OR elevat*))))) NOT TI = (((child* OR adolescen* OR infan* ORnewborn* OR neonat*)) NOT ((adult*))) AND DT = (article) AND LA = (english).

**Cochrane CENTRAL.**

((sepsis OR septic* OR Bacteremia OR Bacteraemia OR fungemia OR fungaemia OR urosepsis OR infection* OR pneumonia* OR meningitis* OR meningoencephalitis*):ab,ti) AND ((((emergency) NEAR/6 (ward* OR department* OR room OR unit OR cent* OR patient* OR care OR healthcare OR physician* OR medicine)) OR ‘acute care’ OR ed):ab,ti) AND ((((biological* OR bio* OR inflammat* OR diagnos* OR molecul* OR disease) NEAR/3 marker*) OR biomarker* OR ((serum OR blood) NEAR/6 (concentrate* OR level* OR marker* OR lactate OR protein* OR elevat*))):ab,ti) NOT (((child* OR adolescen* OR infan* OR newborn* OR neonat*):ti) NOT ((adult*):ti))

**Google scholar.**

sepsis|septic|Bacteremia|Bacteraemia|fungemia|fungaemia|urosepsis|infection|pneumonia|meningitis|meningoencephalitis “emergency ward|department|room”|"acute care” “biological|bio|inflammatory marker|markers”|biomarker|biomarkers -child -infant –newborn.

## Abbreviations

APACHE-2: Acute Physiologic Assessment and Chronic Health Evaluation II
SOFA: Sequential Organ Failure Assessment
AUC: Area under the curve
CRP: C-reactive protein
ED: Emergency department
H-FABT: Heart fatty acid binding protein
IL-6: Interleukin-6
MEDS: Mortality in Emergency Department Sepsis
MMP9: Matrix metallopeptidase 9
NEWS: National Early Warning Score
NGAL: Neutrophil gelatinase-associated lipocalin
PCT: Procalcitonin
PRISMA: Preferred Reporting Items for Systematic Reviews and Meta-analyses
PROBAST: Prediction model Risk Of Bias Assessment Tool
PTX3: Pentraxin 3
qSOFA: Quick Sequential Organ Failure Assessment
SAPS-2: Simplified Acute Physiology Score 2
sPD-1: Soluble programmed death 1
sTM: Soluble thrombomodulin
suPAR: Soluble urokinase-type plasminogen activator receptor
TIMP-1: Tissue inhibitor of metalloproteinase-1
