# Supplementary material for: Predicting mortality in adult patients with sepsis in the emergency department by using combinations of biomarkers and clinical scoring systems: a systematic review
Source: BMC Emerg Med. 2021 Jun 13;21:70. doi: 10.1186/s12873-021-00461-z (PMC8201689; doi:10.1186/s12873-021-00461-z)
Supplement: Supplementary file 1 — Additional file 1: Supplemental Table 1: overview of commonly used clinical scoring systems for mortality prediction. Supplemental table 2: Extended PROBAST checklist. Supplemental table 3: Signaling questions used in PROBAST checklist. [file 12873_2021_461_MOESM1_ESM.docx]

| **Clinical scoring system** | **Number of variables required** | **Indended use** |
| --- | --- | --- |
| qSOFA^9^ | 3 | In-hospital patients with an infection |
| NEWS^13^ | 6 | In-hospital patients |
| MEDS^12^ | 9 | ED patients with sepsis |
| SOFA^9^ | 6 | ICU patients |
| CHARLSON^50^ | 17 | Long term mortality risk in patients with chronic diseases |
| CURB-65^51^ | 5 | Patients with community aquired pneumonia |
| APACHE-IV^52^ | 142 | ICU patients |
| SAPS-2^53^ | 15 | ICU patients |
|  |  |  |
| **Supplemental Table 1: overview of commonly used clinical scoring systems for mortality prediction** | | |
| qSOFA: Quick Sequential Organ Failure Assessment, NEWS: National Early Warning Score, MEDS: Mortality in Emergency Department Sepsis, SOFA: Sequential Organ Failure Assessment, CHARLSON: Charlson Comorbidity Index, APACHE-IV: Acute Physiology And Chronic Health Evaluation-4, SAPS-2: Simplified Acute Physiology Score-2 | | |

| **Author, year** | **Domain 1: participants** | **1.1** | **1.2** | **1B** | **DOMAIN 2: Predictors** | **2.1** | **2.2** | **2.3** | **2B** | **DOMAIN 3: Outcome** | **3.1** | **3.2** | **3.3** | **3.4** | **3.5** | **3.6** | **3B** |
| --- | --- | --- | --- | --- | --- | --- | --- | --- | --- | --- | --- | --- | --- | --- | --- | --- | --- |
| Zhao, 2018 | High | Yes | No | No | Low | Yes | Yes | Yes | No | Low | Yes | Yes | Yes | Yes | Yes | Yes | No |
| Duplessis, 2018 | Low | Yes | Yes | No | Low | Yes | Yes | Yes | No | Low | Yes | Yes | Yes | Yes | Yes | Yes | No |
| Niño, 2017 | Low | Yes | Yes | No | Low | Yes | Yes | Yes | No | Low | Yes | Yes | Yes | Yes | Yes | Yes | No |
| Zhang, 2016 | Low | Yes | Yes | No | Low | Yes | Yes | Yes | No | Low | Yes | Yes | Yes | Yes | Yes | Yes | No |
| Carpio, 2015 | Low | Yes | Yes | No | Low | Yes | Yes | Yes | No | Low | Yes | Yes | Yes | Yes | Yes | Yes | No |
| Zhang, 2014 | Low | Yes | Yes | No | Low | Yes | Yes | Yes | No | Low | Yes | Yes | Yes | Yes | Yes | Yes | No |
| Wang, 2014 | Low | Yes | Yes | No | Low | Yes | Yes | Yes | No | Low | Yes | Yes | Yes | Yes | Yes | Yes | No |
| Chen, 2014 | Low | Yes | Yes | No | Low | Yes | Yes | Yes | No | Yes | Yes | Yes | Yes | Yes | Yes | Yes | No |
| Chen, 2019 | Low | Yes | Yes | No | Low | Yes | Yes | Yes | No | Low | Yes | Yes | Yes | Yes | Yes | Yes | No |
| Zhao, 2013 | Low | Yes | Yes | No | Low | Yes | Yes | Yes | No | Yes | Yes | Yes | Yes | Yes | Yes | Yes | No |
| Kofoed, 2008 | Low | Yes | Yes | No | Low | Yes | Yes | Yes | No | Yes | Yes | Yes | Yes | Yes | Yes | Yes | No |
| Yamamoto, 2015 | Low | Yes | Yes | No | Low | Yes | Yes | Yes | No | Low | Yes | Yes | Yes | Yes | Yes | Yes | No |
| Yin, 2013 | Low | Yes | Yes | No | Low | Yes | Yes | Yes | No | Low | Yes | Yes | Yes | Yes | Yes | Yes | No |
| Liu, 2013 | Low | Yes | Yes | No | Low | Yes | Yes | Yes | No | Low | Yes | Yes | Yes | Yes | Yes | Yes | No |
| Viallon, 2008 | Low | Yes | Yes | No | Low | Yes | Yes | Yes | No | Low | Yes | Yes | Yes | Yes | Yes | Yes | No |
| Henning, 2019 | Low | Yes | Yes | No | Low | Yes | Yes | Yes | No | Low | Yes | Yes | Yes | Yes | Yes | Yes | No |
| Song, 2019 | Low | Yes | Yes | No | Low | Yes | Yes | Yes | No | Low | Yes | Yes | Yes | Yes | Yes | Yes | No |
| Yu, 2019 | Low | Yes | Yes | No | Low | Yes | Yes | Yes | No | Low | Yes | Yes | Yes | Yes | Yes | Yes | No |

**Supplemental table 2:** Extended PROBAST checklist

| **Author, year** | **DOMAIN 4: Analysis** | **4.1** | **4.2** | **4.3** | **4.4** | **4.5** | **4.6** | **4.7** | **4.8** | **4.9** |
| --- | --- | --- | --- | --- | --- | --- | --- | --- | --- | --- |
| Zhao, 2018 | Low | Yes | Yes | Yes | Yes | Yes | Yes | Yes | Yes | Yes |
| Duplessis, 2018 | High | No | Yes | Yes | Yes | Yes | Yes | Yes | No | Yes |
| Niño, 2017 | High | Yes | Yes | Yes | Unknown | Yes | Yes | Yes | No | Yes |
| Zhang, 2016 | Low | Yes | Yes | Yes | Unknown | Yes | Yes | Yes | Yes | Yes |
| Carpio, 2015 | High | No | Yes | Yes | Unknown | Yes | Yes | Yes | No | Yes |
| Zhang, 2014 | High | Yes | Yes | Yes | Unknown | Yes | Yes | Yes | No | Yes |
| Wang, 2014 | High | No | Yes | Yes | Unknown | Yes | Yes | Yes | No | Yes |
| Chen, 2014 | Low | Yes | Yes | Yes | Unknown | Yes | Yes | Yes | Yes | Yes |
| Chen, 2019 | Low | Yes | Yes | Yes | Yes | Yes | Yes | Yes | Yes | Yes |
| Zhao, 2013 | Low | Yes | Yes | Yes | Yes | Yes | Yes | Yes | Yes | Yes |
| Kofoed, 2008 | High | No | Yes | Yes | Unknown | No | Yes | Yes | No | Yes |
| Yamamoto, 2015 | Low | Yes | Yes | Yes | Yes | Yes | Yes | Yes | Yes | Yes |
| Yin, 2013 | High | Yes | Yes | Yes | Unknown | Yes | Yes | Yes | Yes | Yes |
| Liu, 2013 | High | Yes | Yes | Yes | Unknown | Yes | Yes | Yes | Yes | Yes |
| Viallon, 2008 | High | No | Yes | Yes | Unknown | No | Yes | Yes | No | Yes |
| Henning, 2019 | High | No | Yes | No | Yes | No | Yes | Yes | No | Yes |
| Song, 2019 | High | No | Yes | Yes | Unknown | Yes | Yes | Yes | No | Yes |
| Yu, 2019 | Low | Yes | Yes | Yes | Yes | Yes | Yes | Yes | Yes | Yes |

**Supplemental table 2:** Extended PROBAST checklist

| **1.1** | Were appropriate data sources used, e.g. cohort, RCT or nested case-control study data? |
| --- | --- |
| 1.2 | Were all inclusions and exclusions of participants appropriate? |
| 1B | Concern that the included participants and setting do not match the review question |
| 2.1 | Were predictors defined and assessed in a similar way for all participants? |
| 2.2 | Were predictor assessments made without knowledge of outcome data? |
| 2.3 | Are all predictors available at the time the model is intended to be used? |
| 2B | Concern that the definition, assessment or timing of predictors in the model do not match the review question |
| 3.1 | Was the outcome determined appropriately? |
| 3.2 | Was a pre-specified or standard outcome definition used? |
| 3.3 | Were predictors excluded from the outcome definition? |
| 3.4 | Was the outcome defined and determined in a similar way for all participants? |
| 3.5 | Was the outcome determined without knowledge of predictor information? |
| 3.6 | Was the time interval between predictor assessment and outcome determination appropriate? |
| 3B | Concern that the outcome, its definition, timing or determination do not match the review question |
| 4.1 | Were there a reasonable number of participants with the outcome? |
| 4.2 | Were continuous and categorical predictors handled appropriately? |
| 4.3 | Were all enrolled participants included in the analysis? |
| 4.4 | Were participants with missing data handled appropriately? |
| 4.5 | Was selection of predictors based on univariable analysis avoided? |
| 4.6 | Were complexities in the data (e.g. censoring competing risks sampling of controls) accounted for appropriately? |
| 4.7 | Were relevant model performance measures evaluated appropriately? |
| 4.8 | Were model overfitting and optimism in model performance accounted for? |
| 4.9 | Do predictors and their assigned weights in the final model correspond to the results from multivariable analysis? |

**Supplemental table 3:** Signaling questions used in PROBAST checklist

50. Charlson ME, Pompei P, Ales KL, MacKenzie CR. A new method of classifying prognostic comorbidity in longitudinal studies: development and validation. J Chronic Dis. 1987;40(5):373–83. <https://doi.org/10.1016/0021-9681(87)90171-8>.

51. Lim WS, van der Eerden MM, Laing R, Boersma WG, Karalus N, Town GI, et al. Defining community acquired pneumonia severity on presentation to hospital: an international derivation and validation study. Thorax. 2003;58(5):377–82. <https://doi.org/10.1136/thorax.58.5.377>.

52. Zimmerman JE, Kramer AA, McNair DS, Malila FM. Acute physiology and chronic health evaluation (APAC HE) IV: hospital mortality assessment for today's critically ill patients. Crit Care Med. 2006;34(5):1297–310. <https://doi.org/10.1097/01.CCM.0000215112.84523.F0>.

53. Le Gall JR, Lemeshow S, Saulnier F. A new simplified acute physiology score (SAPS II) based on a European/north American multicenter study. JAMA. 1993;270(24):2957–63. <https://doi.org/10.1001/jama.1993.03510240069035>.
